# Tumor vascularity and lipiodol deposition as early radiological markers for predicting risk of disease progression in patients with unresectable hepatocellular carcinoma after transarterial chemoembolization

**DOI:** 10.18632/oncotarget.6892

**Published:** 2016-01-12

**Authors:** Cheng-Shi Chen, Fang-Kun Li, Chen-Yang Guo, Jin-Cheng Xiao, Hong-Tao Hu, Hong-Tao Cheng, Lin Zheng, Deng-Wei Zong, Jun-Li Ma, Li Jiang, Hai-Liang Li

**Affiliations:** ^1^ Department of Radiology, Zhengzhou University Affiliated Cancer Hospital, Zhengzhou 450008, China

**Keywords:** hepatocellular carcinoma, transarterial chemoembolization, lipiodol, tumor vascularity, overall survival

## Abstract

This study evaluated the factors impacting overall survival (OS) and time to progression (TTP) in patients with unresectable hepatocellular carcinoma (HCC) who received transarterial chemoembolization (TACE). HCC patients were grouped based on tumor vascularity and lipidiol deposition after TACE. Tumor vascularity was classified based on contrast enhancement on arterial phase baseline CT scans. Lipiodol deposition was evaluated using CT scans. The progression-free rate was significantly higher in patients with good blood supply + good lipiodol deposition compared to those with good blood supply + poor lipiodol deposition. In patients with poor lipidiol deposition, risk of death was significantly positively correlated with stage, and negatively correlated with number of TACE procedures and degree of lipidiol deposition after the first TACE. Risk of disease progression in these patients was positively correlated with tumor size, and negatively correlated with number of TACE procedures and degree of lipidiol deposition after the first TACE. Our data showed that tumor vascularity and lipiodol deposition can be used as early radiological markers to identify patients who do not respond to TACE, and who can be considered earlier for alternative combination treatment strategies. Our data also indicated that poor lipiodol retention may predict a poor TTP and OS despite the blood supply status.

## INTRODUCTION

Hepatocellular carcinoma (HCC) is the most common malignancy of the liver, and the third leading cause of cancer-related deaths worldwide [1]. Chronic infection with the hepatitis B virus (HBV) or hepatitis C virus (HCV) are the most important risk factors for HCC, and patients with untreated, unresectable HCC have a median survival time of < 6 months [2, 3]. Almost 75% of all HCC cases are seen in Asia [4], and HCC is a leading cause of cancer-related deaths in China [5].

Current therapeutic options are based on the Barcelona Clinic Liver Cancer (BCLC) staging system which integrates tumor characteristics and performance status with liver function [6]. Surgical resection and liver transplantation is the currently accepted treatment of choice in patients who have early stage HCC with decompensated cirrhosis [7, 8]. However, a majority of HCC patients present at the intermediate (BCLC stage B) or advanced (BCLC stage C) stage of disease. Patients with BCLC stage B are currently treated with transarterial chemoembolization (TACE) [9]. TACE, which selectively obstructs the tumor vessels, is conventionally performed by intra-arterial infusion of a viscous emulsion of an ethiodized oil such as lipiodol, along with a chemotherapeutic agent. This is followed by an injection of gelatin sponge particles which embolize the blood vessel [10]. Embolization ensures the retention of lipiodol thereby enhancing drug delivery, reducing drug washout from the tumor, and inducing ischemic necrosis. Molecular targeted drugs such as sorafenib have also been recently used to treat patients with unresectable/metastatic disease who are unresponsive to conventional therapy [11].

A number of studies have investigated the prognostic value of lipiodol retention and tumor vascularity in HCC. Although early studies did not support the hypothesis that lipiodol deposition was a predictor of overall survival (OS) [12], other studies showed that HCC tumors which did not exhibit lipiodol retention had a lower treatment efficacy and higher risk of liver damage [13–16]. Furthermore, TACE was found to be as effective as hepatic resection when lipiodol was compactly retained within the tumor [17]. Additionally, patients with well-differentiated, hypovascular HCC tumors responded poorly to TACE, had a shorter OS, and were associated with early recurrence compared to patients with well-differentiated hypervascular tumors [18, 19, 20, 21]. However, in patients with unresectable HCC treated with TACE, OS was higher in hypervascular responders, and in hypervascular non-responders and hypovascular responders compared to hypovascular non-responders [3]. These data strongly suggested that tumor vascularity is not the only determinant of treatment outcome in TACE-treated HCC patients.

The prognostic significance of biomarkers such as alpha-fetoprotein, alpha-fetoprotein-L3 and des-carboxy-prothrombin after TACE remains unclear [22]. However, angiogenesis is known to be key to the pathophysiology of HCC, and increased serum vascular endothelial growth factor-A (VEGF-A) and angiopoietin-2 levels after TACE were shown to predict rapid HCC growth [23]. Similarly, increased levels of serum VEGF levels 1-2 days after TACE in HCC patients was shown to be associated with distant metastasis and poor outcomes [24]. There is a current imperative need to identify novel and reliable targets and biomarkers which would allow the clinician to predict the course of HCC post-treatment in order to personalize therapeutic strategies.

Based on current EASL guidelines, TACE is ruled ineffective only after a patient goes through two or three cycles of TACE with progressing disease. Our present study aims to identify early markers that can predict responsiveness to TACE treatment after the first TACE procedure. Identification of early markers is important because patients who do not respond to TACE can be considered earlier for alternative combination treatment strategies instead of waiting for two or three cycles of TACE. In this study, we evaluated the prognostic value of tumor vascularity and lipiodol deposition on OS and time to progression (TTP) in HCC patients treated with TACE. We also investigated the impact of other risk factors including baseline tumor size, number of nodules, Child Pugh score, blood supply, AFP, gender, age, BCLC stage, and ECOG score on OS and TTP.

## RESULTS

### Baseline characteristics of subjects with good blood supply

There were 88 males and 13 females in the group with good blood supply. The patients had a mean age of 57.35 years. There were 63 patients who were BCLC stage B and 38 patients who were BCLC stage C. The mean tumor size was 8.2 cm, the mean number of nodules was 3.05, the mean Child Pugh score was 5.54, and the mean baseline AFP was 614.74. The study patients had a mean ECOG Performance Status of 0.45, and the mean number of times they had received TACE was 2.74. The mean degree of lipiodol deposition after the first TACE was 58.32 %. A total of 59 subjects had tumors with a good blood supply + good lipiodol deposition, and 42 subjects had tumors with a good blood supply + poor lipiodol deposition. The mean survival time was 31.44 months and the mean time to disease progression was 11.8 months (Table 1).

**Table 1:** Baseline characteristics of subjects with good blood supply and with poor lipiodol deposition

	Subjects with good blood supply (*N* = 101)	Subjects with poor lipiodol deposition (*N* =73)
Gender		
Male	88(87.13%)	62(84.93%)
Female	13(12.87%)	11(15.07%)
Age (years)	57.35±11.71	57.01±12.01
Tumor size (cm)	8.2±4.63	8.41±5.11
Number of nodules	3.05±3.66	3±2.75
Child Pugh score	5.54±0.88	5.44±0.86
AFP	614.74±561.79	598.82±534.36
ECOG performance status	0.45±0.5	0.52±0.5
No. of TACE procedures	2.74±1.19	2.49±0.97
BCLC stage		
B	63(62.38%)	40(54.79%)
C	38(37.62%)	33(45.21%)
Degree of lipiodol deposition after 1^st^ TACE (%)	58.32±25.92	26.15±12.93
Good lipiodol deposition (≥50%)	59(58.42%)	
Poor lipiodol deposition (< 50 %)	42(41.58%)	
Tumor blood supply		
poor blood supply		31(42.47%)
good blood supply		42(57.53%)
Survival time (months)	31.44±25.91	28.68±30.57
Disease progression time (months)	11.8±11.67	9.34±12.96

### Univariate and multivariate analysis to determine factors associated with OS in subjects with good blood supply

Univariate analyses showed that tumor size, AFP, number of TACE procedures, BCLC stage, and the degree of lipiodol deposition after the 1^st^ TACE procedure were significantly associated with OS (p < 0.05). The hazard of death was increased 1) with every cm increase in tumor size (HR=1.05, *p* = 0.017); 2) with every unit increase in AFP levels (HR=1.0004, *p* = 0.041); and 3) in subjects with BCLC stage C compared to BCLC stage B (HR=3.23, p < 0.001). The hazard of death was decreased 1) with each increase in the number of TACE procedures (HR=0.65, p < 0.001); and 2) with each percent increase in the degree of lipiodol deposition (HR=0.99, *p* = 0.024).

**Figure 1:**
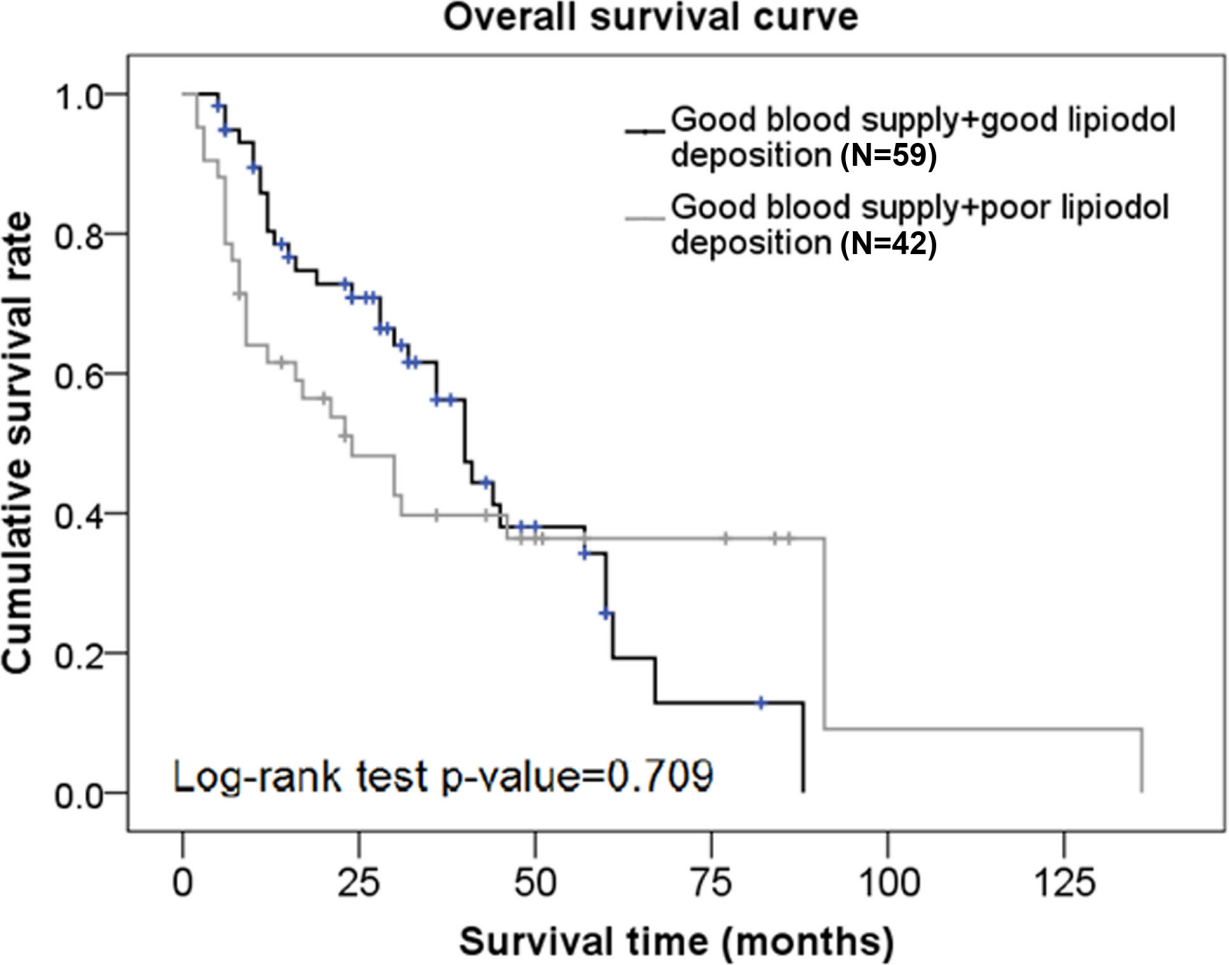
Overall survival curves in subjects from the good blood supply group who had different degrees of lipiodol deposition

Factors that were significantly associated with OS on univariate analysis were included in the multivariate analysis. After adjusting for all other factors, data from the multivariate analysis showed that the hazard of death was decreased with each increase in the number of TACE procedures (HR=0.62, *p* = 0.002); and was increased in subjects with BCLC stage C compared to BCLC stage B (HR=2.6, *p* = 0.001) (Table 2). There was no significant difference in OS rates between subjects with good blood supply + good lipiodol deposition and those with good blood supply + poor lipiodol deposition (*p* = 0.709) (Figure 1).

**Table 2:** Univariate and multivariate analysis to determine factors associated with OS and disease progression in subjects with good blood supply

	Univariate		Multivariate		Multivariate 2	
	HR (95%CI)	*p*-value	HR (95%CI)	*p*-value	HR (95%CI)	*p*-value
**For overall survival**						
Gender						
Male	1.45(0.58-3.63)	0.432				
Female	ref					
Age	0.999(0.98-1.02)	0.943				
Tumor size	1.05(1.01-1.09)	0.017*	1.02(0.97-1.08)	0.432		
Number of nodules	1.05(0.997-1.11)	0.062				
Child Pugh score	1.14(0.88-1.49)	0.324				
AFP	1.0004(1.00002-1.001)	0.041*	1.0003(0.9998-1.001)	0.244		
ECOG performance status	1.36(0.87-2.13)	0.184				
TACE times	0.65(0.51-0.82)	<0.001*	0.62(0.46-0.83)	0.002*		
BCLC stage						
B	ref		ref			
C	3.23(1.91-5.45)	<0.001*	2.6(1.49-4.52)	0.001*		
Degree of lipiodol deposition after 1^st^ TACE (%)	0.99(0.98-0.999)	0.024*	0.997(0.99-1.01)	0.555		
Good lipiodol deposition (≥50%)	0.91(0.54-1.53)	0.713				
Poor lipiodol deposition (<50 %)	ref					
**For disease progression**						
Gender						
Male	0.48(0.26-0.88)	0.017*	0.66(0.35-1.25)	0.206	0.64(0.34-1.2)	0.164
Female	ref		ref		ref	
Age	0.99(0.97-1.01)	0.39				
Tumor size	1.03(0.99-1.07)	0.204				
Number of nodules	1.12(1.06-1.19)	<0.001*	1.12(1.05-1.19)	0.001*	1.13(1.06-1.2)	<0.001*
Child Pugh score	1.19(0.96-1.49)	0.119				
AFP	1.0002(0.9999-1.001)	0.241				
ECOG performance status	1.94(1.29-2.92)	0.002*	1.59(1.03-2.44)	0.036*	1.65(1.07-2.53)	0.022*
TACE times	0.97(0.82-1.15)	0.763				
BCLC stage						
B	ref					
C	1.1(0.73-1.65)	0.662				
Degree of lipiodol deposition after 1^st^ TACE (%)	0.99(0.98-0.99)	0.001*	0.99(0.98-0.999)	0.025*		
Good lipiodol deposition (≥50%)	0.65(0.44-0.98)	0.037*			0.73(0.48-1.11)	0.139
Poor lipiodol deposition (<50%)	ref				ref	

* p < 0.05, significant associated with OS or disease progression OS, Overall Survival

### Univariate and multivariate analysis to determine factors associated with disease progression in subjects with good blood supply

Univariate analyses showed that gender, number of nodules, ECOG Performance Status, the degree of lipiodol deposition after the first TACE, and lipiodol deposition ≥ 50% were significantly associated with disease progression (p < 0.05). The hazard of disease progression was increased 1) with increasing number of nodules (HR=1.12, p < 0.001); 2) with every increase in the ECOG Performance status (HR=1.94, *p* = 0.002). The hazard of disease progression was significantly decreased 1) in males compared to females (HR=0.48, *p* = 0.017); and 2) with every percent increase in the degree of lipiodol deposition (HR=0.99, *p* = 0.001); 3) in subjects with good lipiodol deposition (≥ 50%) compared to those with poor lipiodol deposition (< 50%) (Table 2).

Factors significantly associated with disease progression by univariate analysis were included in the multivariate analysis. After adjusting for all other factors, data from the multivariate analysis showed that the hazard of disease progression was increased with 1) increasing number of nodules (HR=1.12, *p* = 0.001); and 2) every increase in the ECOG Performance status (HR=1.59, *p* = 0.036). The hazard of disease progression was decreased with every percent increase in the degree of lipiodol deposition after the first TACE procedure (HR=0.99, *p* = 0.025) (Multivariate 1, Table 2).

Patients were categorized into two groups based on their lipiodol deposition (< 50% lipiodol deposition or > 50% lipiodol deposition). After adjusting for all other factors, the lipiodol deposition group was not associated with disease progression (p > 0.05) (Multivariate 2, Table 2).

**Figure 2:**
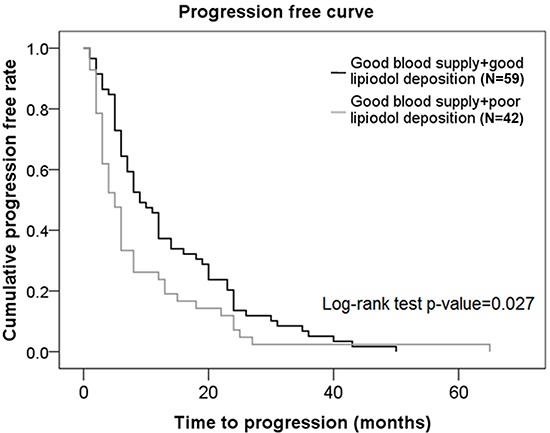
Progression-free curves in subjects from the good blood supply group who had different degrees of lipiodol deposition

Patients with good blood supply + good lipiodol deposition had a significantly higher progression-free rate compared to patients with good blood supply + poor lipiodol deposition (*p* = 0.027) (Figure 2).

### Baseline characteristics of subjects with poor lipiodol deposition

There were 62 males and 11 females in the group with poor lipiodol deposition. Patients in this group had a mean age of 57.01 years. There were 40 subjects who were BCLC stage B, and 33 subjects who were BCLC stage C. The mean tumor size was 8.41 cm, the mean number of nodules was 3, the mean Child Pugh score was 5.44, and the mean baseline AFP was 598.82. The study patients had a mean ECOG Performance Status of 0.52, and the mean number of times they had received TACE was 2.49. The mean degree of lipiodol deposition after the first TACE was 26.15 %. A total of 42 subjects had tumors with good blood supply, and 31 subjects had tumors with poor blood supply. The mean survival time was 28.68 months and the mean time to disease progression was 9.34 months (Table 1).

### Univariate and multivariate analysis to determine factors associated with OS in subjects with poor lipiodol deposition

Univariate analyses showed that tumor size, number of TACE procedures, BCLC stage, and the degree of lipiodol deposition after the 1^st^ TACE procedure were significantly associated with OS (p < 0.05). The hazard of death was increased 1) with every cm increase in tumor size (HR=1.05, *p* = 0.049); and 2) in subjects with BCLC stage C compared to those with BCLC stage B (HR=3.74, p < 0.001). The hazard of death was decreased 1) with each increase in the number of TACE procedures (HR=0.49, *p* = 0.002); and 2) with each percent increase in the degree of lipiodol deposition (HR=0.97, *p* = 0.019) (Table 3).

**Table 3:** Univariate and multivariate analysis to determine factors associated with OS and disease progression in subjects with poor lipiodol deposition

	Univariate		Multivariate	
	HR (95%CI)	*p*-value	HR (95%CI)	*p*-value
**For overall survival**				
Gender				
Male	1.08(0.45-2.56)	0.863		
Female	ref			
Age	1.02(0.99-1.05)	0.154		
Tumor size	1.05(1.0003-1.1)	0.049*	1.02(0.96-1.1)	0.506
Number of nodules	0.999(0.89-1.12)	0.981		
Child Pugh score	1.03(0.69-1.54)	0.878		
AFP	1.001(0.9999-1.001)	0.078		
ECOG performance status	1.2(0.66-2.19)	0.549		
TACE times	0.49(0.32-0.77)	0.002*	0.52(0.34-0.79)	0.002*
BCLC stage				
B	ref		ref	
C	3.74(1.93-7.25)	<0.001*	3.56(1.69-7.52)	0.001*
Degree of lipiodol deposition after 1^st^ TACE (%)	0.97(0.95-0.995)	0.019*	0.97(0.94-0.99)	0.015*
Tumor blood supply				
poor blood supply	ref			
good blood supply	0.999(0.55-1.83)	0.997		
**For disease progression**				
Gender				
Male	0.72(0.37-1.39)	0.331		
Female	ref			
Age	1.01(0.99-1.03)	0.209		
Tumor size	1.04(1.004-1.08)	0.032*	1.05(1.01-1.1)	0.023*
Number of nodules	1.04(0.95-1.13)	0.386		
Child Pugh score	1.39(1.07-1.8)	0.015*	1.22(0.93-1.6)	0.154
AFP	1.0003(0.9998-1.001)	0.266		
ECOG performance status	1.61(0.99-2.6)	0.053		
TACE times	0.75(0.58-0.98)	0.034*	0.76(0.59-0.99)	0.040*
BCLC stage				
B	ref			
C	1.02(0.63-1.66)	0.928		
Degree of lipiodol deposition after 1^st^ TACE (%)	0.97(0.95-0.99)	0.005*	0.97(0.95-0.996)	0.018*
Tumor blood supply				
poor blood supply	ref			
good blood supply	0.96(0.6-1.55)	0.874		

* p < 0.05, significantly associated with OS or disease progression OS, Overall Survival

**Figure 3:**
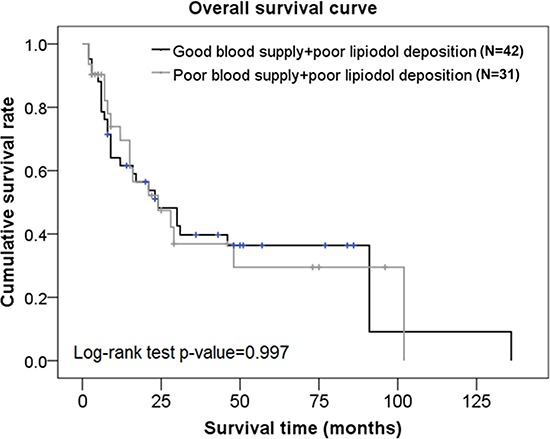
Overall survival curves in subjects from the poor lipiodol deposition group who had different blood supplies

Factors that were significantly associated with OS on univariate analysis were included in the multivariate analysis. After adjusting for all other factors, data from the multivariate analysis showed that the hazard of death was decreased with 1) each increase in the number of TACE procedures (HR=0.52, *p* = 0.002), and 2) with each percent increase in the degree of lipiodol deposition (HR=0.97, *p* = 0.015); and increased in subjects with BCLC stage C compared to those with BCLC stage B (HR=3.56, *p* = 0.001) (Table 3). There was no significant difference in OS rates between subjects with good blood supply + poor lipiodol deposition and those with poor blood supply + poor lipiodol deposition (*p* = 0.997) (Figure 3).

### Univariate and multivariate analysis to determine factors associated with disease progression in subjects with poor lipiodol deposition

Univariate analyses showed that tumor size, Child Pugh score, number of TACE procedures, and the degree of lipiodol deposition after the first TACE were significantly associated with disease progression (p < 0.05). The hazard of disease progression was increased 1) with increasing tumor size (HR=1.04, *p* = 0.032); 2) with every increase in the Child Pugh score (HR=1.39, *p* = 0.015). The hazard of disease progression was significantly decreased 1) with each increase in the number of TACE procedures (HR=0.75, *p* = 0.034); and 2) with every percent increase in the degree of lipiodol deposition (HR=0.97, *p* = 0.005) (Table 3).

Factors significantly associated with disease progression by univariate analysis were included in the multivariate analysis. After adjusting for all other factors, data from the multivariate analysis showed that the hazard of disease progression was increased with increasing tumor size (HR=1.05, *p* = 0.023), and was decreased 1) with each increase in the number of TACE procedures (HR=0.76, *p* = 0.04), and 2) with every percent increase in the degree of lipiodol deposition after the first TACE procedure (HR=0.97, *p* = 0.018) (Table 3).

**Figure 4:**
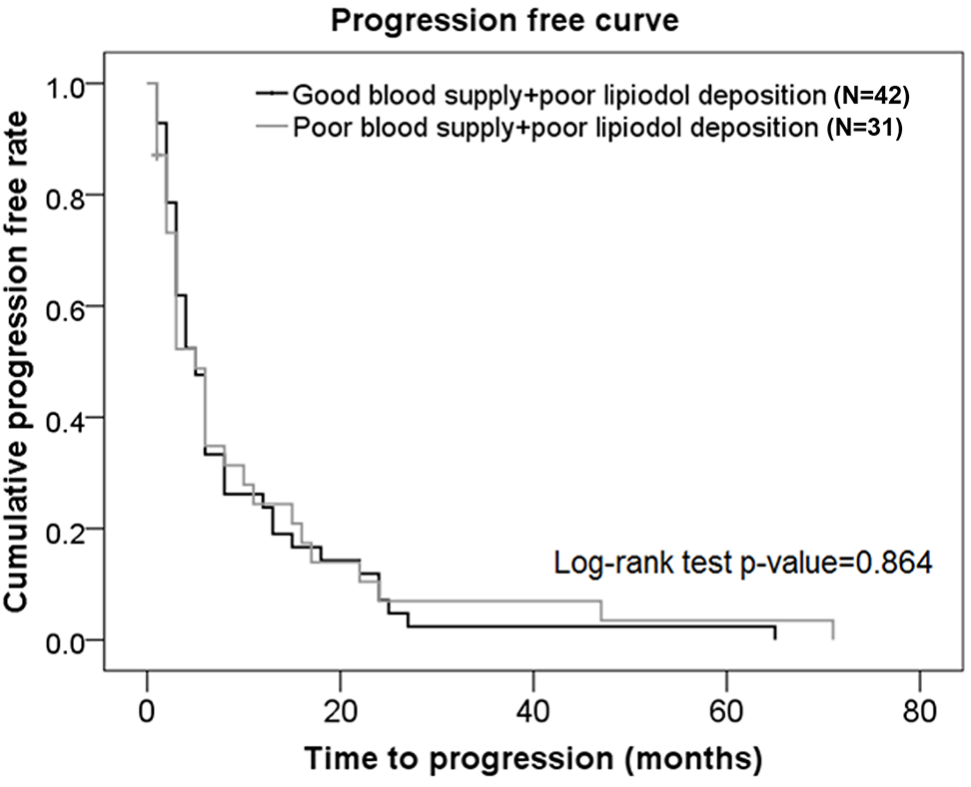
Progression-free curves in subjects from the poor lipiodol deposition group who had different blood supplies

There was no significant difference in progression-free rate between subjects with good blood supply + poor lipiodol deposition and those with poor blood supply + poor lipiodol deposition (*p* = 0.864) (Figure 4).

Representative cases with varied tumor vascularity and lipiodol retention patterns were studied to evaluate if clinical outcomes could be predicted by lipiodol deposition patterns and tumor vascularity. Case 1 was a 56-year old BCLC stage C male patient in whom a good blood supply and poor lipiodol retention predicted a poor TACE outcome (Supplementary Figure S1). Case 2 was a 45-year-old BCLC stage B male patient with poor tumor vascularity, who exhibited a poor lipiodol retention pattern as predicted (Supplementary Figure S2). Case 3 was a 50-year-old BCLC stage C female HCC patient who showed a very good lipiodol retention pattern immediately after TACE, and had a significant decrease in lipiodol retention later with a poor TACE outcome (Supplementary Figure S3). The decrease in lipiodol retention could be either because 1) although the lipiodol retention was originally good, it was gradually degraded by the tumor cells, or 2) it was originally a bad lipiodol retention. Additionally, different situations may call for different treatment modalities. For example, in the first situation, TACE may be performed repeatedly and sorafenib may be used during the interval between TACE sessions; and in the 2nd situation (or in tumors with poor blood supplies), TACE may be stopped relatively early, and sorafenib may be used to delay tumor progression.

## DISCUSSION

In this study, we evaluated the prognostic value of tumor vascularity and lipiodol deposition pattern on OS and TTP in patients with advanced HCC treated with TACE. We showed that tumor vascularization and lipiodol deposition can be used as radiological markers to predict/reflect treatment efficacy after TACE. A radiological finding of angiogenesis after TACE at the site of the tumor is a novel marker for early identification of TACE non-respondent patients, who can then be treated with alternative combination treatment strategies.

A number of studies have investigated factors that impact disease progression and survival after TACE in patients with advanced HCC. Tumor number > 5 was shown to be a significant negative predictor of outcome after TACE [21]. Our data were consistent with this study and showed that increased number of nodules correlated with a significantly higher risk of disease progression in the good blood supply group. Although Child-Pugh grading was previously shown to be an independent predictor of overall survival [25, 26], we did not observe a correlation between Child Pugh score and OS or disease progression in the two groups of patients. Advanced disease stage (BCLC stage C) has previously been shown to be a negative predictor for TACE in randomized controlled trials [27], and this agreed with our present results which showed a higher risk of death in BCLC stage C patients compared to BCLC stage B patients in both study groups. We showed an inverse correlation between hazard of death and number of TACE cycles in all patients. Additionally, patients with poor lipiodol deposition had a decreased hazard of disease progression with increasing number of TACE procedures. Our data were consistent with previous results showing a positive correlation between number of TACE cycles and OS and disease progression [28].

Angiogenesis is thought to influence tumor survival after TACE likely via the VEGF signaling pathway [29]. Studies investigating tumor vascularity as a factor impacting treatment outcome after TACE showed that high serum VEGF levels were significantly correlated with poor outcomes after TACE [30]. However, other studies showed that patients with hypovascular HCC had a poorer response, poorer survival, and early recurrence compared to patients with hypervascular tumors [3, 18, 20, 21], possibly because the chemotherapeutic agent or embolic agent is more efficiently delivered to tumors with a good arterial supply. These data suggested that the role of tumor vascularity in treatment response and OS is not completely understood. Lipiodol accumulation has also been suggested as an important prognostic factor after TACE, although the mechanism remains unclear. It was recently shown that uptake of non-compact lipiodol was a risk factor for local recurrence, and that uptake of compact lipiodol resulted in complete necrosis of resected tissue [31, 32]. In HCC patients with portal vein tumor thrombus (PVTT), lipiodol accumulation in the PVTT after TACE was a predictor of longer survival [26]. TACE-treated HCC patients who had lipiodol accumulation surrounding the HCC lesion were shown to have significantly higher disease free survival (DFS) rates compared to patients with lipiodol accumulation involving the lesion, or covering the lesion [33].

Using a combination of tumor vascularity as well as lipiodol deposition as a prognostic factor for TACE, we showed that although there was no significant difference in OS rates between subjects with good blood supply + good lipiodol deposition and those with good blood supply + poor lipiodol deposition, the progression-free rates were significantly higher in patients with good blood supply + good lipiodol deposition compared to those with good blood supply + poor lipiodol deposition. There was no significant difference in OS rates or progression-free rates between subjects with good blood supply + poor lipiodol deposition and those with poor blood supply + poor lipiodol deposition. Interestingly, patients with good tumor vascularity are generally considered to have good lipiodol retention and therefore, a good prognosis. This was demonstrated in our representative Case 3 patient who showed angiogenesis at the original site of the tumor, and a good lipiodol retention pattern immediately after TACE. However, although this patient had a good TACE outcome initially, there was a significant decrease in lipiodol retention at a later stage leading to a poor TACE outcome in this patient. These data suggested that a poor lipiodol retention would result in a poor TACE outcome despite the status of tumor vascularity.

Previous studies showed that TACE induced the expression of angiogenic factors in residual tumor cells. Part of the non-embolized liver exhibited compensatory hyperplasia, and HCC recurrence was attributed to the establishment of collateral circulation after TACE. It was thought that this could play an important role in reducing lipiodol deposition [34, 35]. These data support our hypothesis that 1) tumor vascularity as well as lipiodol deposition should be considered during clinical management after TACE, and 2) a poor lipiodol retention pattern would result in a poor TACE outcome, despite the blood supply status. Possible reasons for poor lipiodol deposition in tumor may be: 1) Clinicians sometimes fail to find the supply arteries, or in situations of arteriovenous fistula, there is poor blood supply or collateral circulation formation leading to poor lipiodol retention, or 2) Tumor cells “eat” up the deposited lipiodol possibly by remaining in a differentiated state and retaining their ability to consume foreign bodies (Supplementary Figure S3).

In conclusion, current EASL guidelines dictate that TACE non-responsiveness should be determined only after several cycles of TACE. Our present study used tumor vascularity and lipiodol deposition as early radiological markers of TACE non-responsiveness in patients with advanced HCC. We showed that risk of death decreased with increased number of TACE procedures, and increased deposition of lipiodol, while it was increased in subjects with BCLC stage C compared to BCLC stage B. The risk of disease progression was increased with 1) increasing number of nodules and 2) increasing ECOG score. Risk of disease progression was decreased with increasing number of TACE procedures, and with every percent increase in the degree of lipiodol deposition after the first TACE procedure. To the best of our knowledge, this is the first report which used both tumor vascularity and lipiodol deposition to evaluate the risk of disease progression after TACE in advanced HCC. We demonstrated that poor lipiodol retention resulted in poor TACE outcomes, despite good tumor vascularity. In addition, our results may also explain why patients who respond well to TACE do not benefit from addition of sorafenib to their TACE procedure compared to patients who do not respond to TACE. The major limitation of this study was its retrospective nature. Other important limitations are 1) the small sample size, 2) determination of lipiodol deposition is subjective, and 3) although TACE impacts blood supply to the tumor, direct assessment of the tumor is still a challenge.

Our data have important implications for the clinical management of patients with advanced HCC.

## MATERIALS AND METHODS

This retrospective, single-center study evaluated records of successive subjects who were diagnosed with HCC at the Department of Interventional Radiology of Henan Tumor Hospital between April 2004 and March 2012. Inclusion criteria were 1) diagnosis of HCC (BCLC stage B or C); 2) Child-Pugh grade A or B; 3) ECOG score of 0 or 1; 4) received at least two cycles of TACE. Exclusion criteria were 1) previous treatment with microwave ablation, radiofrequency ablation, surgical resection or liver transplantation after TACE; 2) platelet count < 50 × 10^9^ / L. Doxorubicin (Pude Pharmacy, Datong, China) was used at a concentration of 10 – 30 mg/m2 and mixed with 5 – 20 mL of iodized oil (Lipiodol Ultrafluide, Laboratoire Guerbet, Aul-nay-sous-Bois, France). Under fluoroscopic monitoring, the mixture was infused at a rate of 1 – 2 mL/min through a microcatheter until stasis flow in tumor vascularity was achieved. A gelatin sponge (Jingling, Nanjing, China) was used to embolize the feeding artery of the tumor. All treatments in this study were based on the standard of care in alignment with international standards. Follow-up visits were done every 2-3 months. The study was approved by the Ethics Committee of the Henan Tumor Hospital, and written, informed consent was obtained from all study subjects.

Since the inclusion criteria stated that all study patients were required to undergo at least two cycles of TACE, and TACE is the standard therapy for stage B patients, our cohort had a larger number of BCLC stage B patients than BCLC stage C patients. The gender ratio of our study population was typical of the Chinese population. Patients were classified into two groups based on tumor vascularity and lipiodol deposition after the first TACE. There were 101 patients in the good blood supply group and 73 patients in the poor lipiodol deposition group. Tumor vascularity was evaluated as previously described [20, 21]. The degree of contrast enhancement on arterial phase baseline CT scans was used to classify tumors as hypervascular or hypovascular. In hypervascular tumor lesions, the density of tumor lesions was significantly higher than the density of the surrounding liver parenchyma on the enhanced CT arterial phase scan. In hypovascular tumors, the tumor lesion density was equal to or lower than the density of the surrounding liver parenchyma.

Lipiodol deposition was evaluated using CT scans. Areas with lipiodol deposition had a significantly higher density than areas of no lipiodol deposition, while the surrounding liver parenchyma showed medium density.

### Statistical analysis

For baseline distribution, continuous variables were presented as means and standard deviations, and categorical variables were presented as counts and percentages.

Univariate and multivariate Cox proportional hazard models were used to investigate the factors associated with overall survival and disease progression. Factors which were found to be significantly associated with overall survival or disease progression in the univariate model were included in the multivariate analysis. Kaplan-Meier curves were used to determine the survival rates and progression-free rates in subjects in the different experimental groups.

All statistical analyses were performed with the IBM SPSS statistical software version 22 for Windows (IBM Corp., Armonk, New York, USA). A two-tailed *p* of < 0.05 was considered significant.

## SUPPLEMENTARY FIGURES


