# Supplementary material for: Tumor vascularity and lipiodol deposition as early radiological markers for predicting risk of disease progression in patients with unresectable hepatocellular carcinoma after transarterial chemoembolization
Source: Oncotarget. 2016 Jan 12;7(6):7241–52. doi: 10.18632/oncotarget.6892 (PMC4872782; doi:10.18632/oncotarget.6892)
Supplement: Supplementary file 1 [file oncotarget-07-7241-s001.pdf]

SUPPLEMENTARY FIGURES

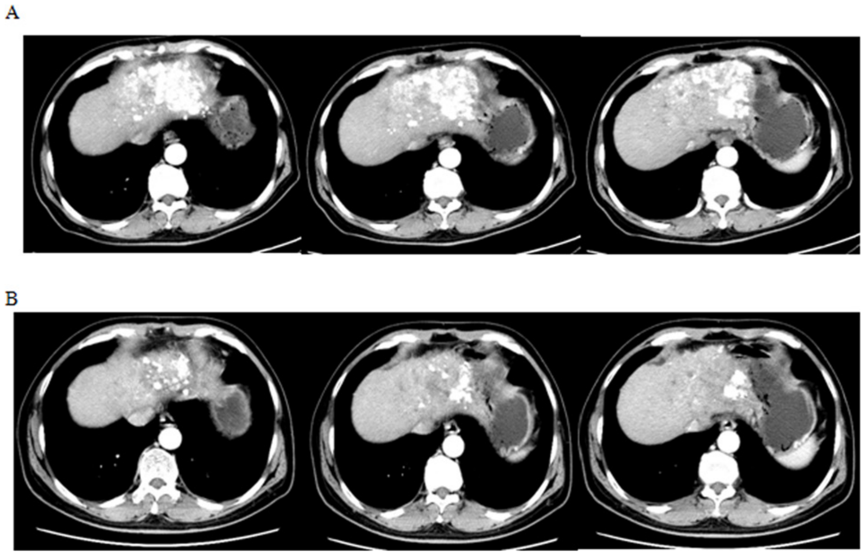

Supplementary Figure S1: A 56-year old BCLC stage C male HCC patient **A.** One month after TACE, the enhanced CT (arterial phase) showed a good lipiodol deposition pattern; **B.** Two months after TACE, the enhanced CT (arterial phase) showed a significant decrease in lipiodol deposition.

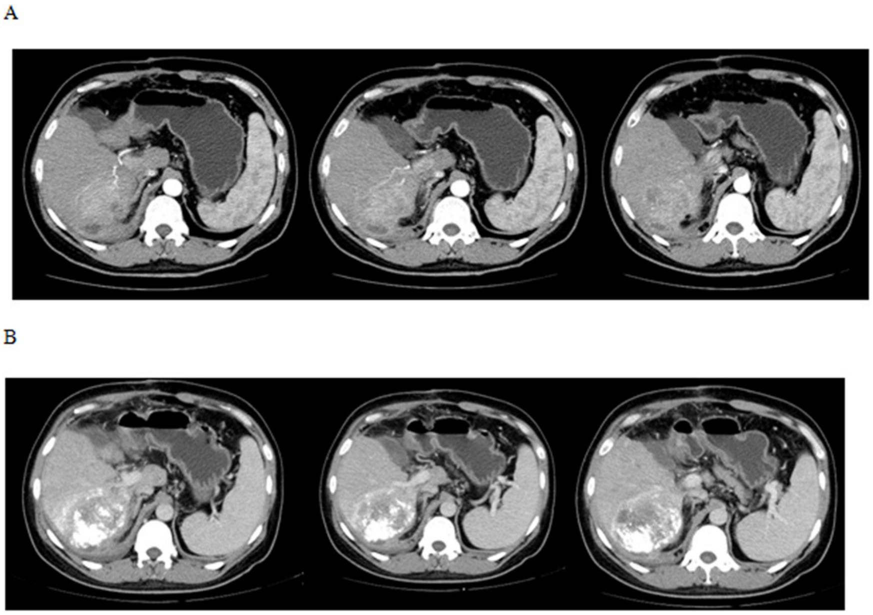

Supplementary Figure S2: A 45-year old stage B male HCC patient **A.** Two weeks before TACE treatment, the enhanced CT (arterial phase) showed significant lesions in the right lobe of the liver and good blood supply; **B.** Four days after TACE, the enhanced CT (portal phase) showed poor lipiodol deposition.

A

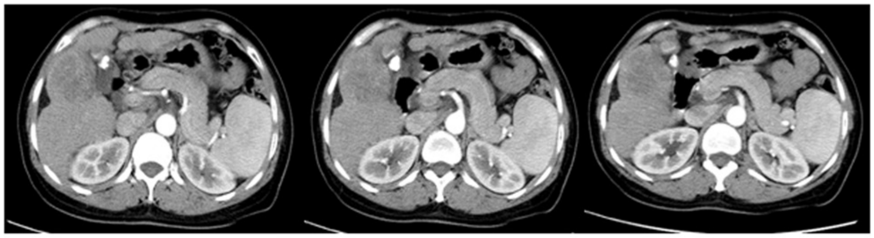

B

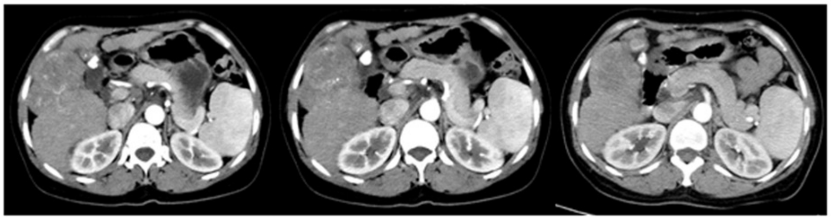

Supplementary Figure S3: A 50-year old stage C female HCC patient **A.** Three days before TACE, the enhanced CT (arterial phase) showed that the enhancement in the right lobe lesion was not obvious, and the blood supply was not abundant; **B.** One month after TACE, the enhanced CT (arterial phase) showed poor lipiodol deposition in the lesion.
